# Impact of lesion preparation and stent optimisation on lesion-oriented events in PCI with drug-eluting stents: 5-year results from the AIDA trial

**DOI:** 10.1007/s12471-025-01937-4

**Published:** 2025-03-06

**Authors:** Mick P. L. Renkens, Maik J. D. Grundeken, Laura S. M. Kerkmeijer, Robin P. Kraak, Deborah N. Kalkman, Rene J. van der Schaaf, Sjoerd. H. Hofma, Karin E. K. Arkenbout, Auke P. J. D. Weevers, Karel T. Koch, Yoshinobu Onuma, Patrick W. Serruys, Jan G. P. Tijssen, Robbert J. de Winter, Joanna J. Wykrzykowska, Ruben Y. G. Tijssen

**Affiliations:** 1 Department of Clinical and Experimental Cardiology, Heart Centre, Amsterdam UMC, University of Amsterdam, Amsterdam, The Netherlands; 2 Department of Cardiology, Isala Heart Centre, Zwolle, The Netherlands; 3 Department of Cardiology, Onze Lieve Vrouwe Gasthuis, Amsterdam, The Netherlands; 4 Department of Cardiology, Medical Centre Leeuwarden, Leeuwarden, The Netherlands; 5 Department of Cardiology, Tergooi Hospital, Blaricum, The Netherlands; 6 Department of Cardiology, Albert Schweitzer Hospital, Dordrecht, The Netherlands; 7 Department of Cardiology, University of Galway, Galway, Ireland; 8 CORRIB Research Centre for Advanced Imaging and Core Laboratory, University of Galway, Galway, Ireland; 9 Department of Cardiology, Thorax Centre, University Medical Centre Groningen, Groningen, The Netherlands; 10 Department of Cardiology, St. Antonius Hospital, Nieuwegein, The Netherlands

**Keywords:** Coronary artery disease, Percutaneous coronary intervention, Implantation strategies, Lesion-oriented events, Revascularisation

## Abstract

**Background:**

Meticulous implantation strategies (i.e. lesion predilatation, stent sizing and postdilatation) are known to decrease lesion-oriented adverse events (LOCE) following percutaneous coronary intervention (PCI) with bioresorbable scaffolds. Their impact on PCI with drug-eluting stents remains unclear.

**Objective:**

To assess the impact of meticulous implantation strategies on long-term LOCE in PCI with everolimus-eluting stents (EES).

**Methods:**

This substudy of the AIDA trial (NCT01858077) focused on the evaluation of predilatation, stent sizing and postdilatation through analyses of vessel and device diameters at various locations around the lesion. Their interrelations were assessed using quantitative coronary angiography across various lesion locations. Logistic regression was used to evaluate how predictors influenced the primary outcome LOCE, which includes target lesion revascularisation (TLR), target-vessel myocardial infarction (TV-MI) and definite stent thrombosis (ST).

**Results:**

LOCE occurred in 84 (7.7%) of 1098 lesions, mainly driven by TLR (63, 5.7%) and TV-MI (46, 4.2%), with ST occurring in 9 (0.8%) lesions. Predilatation and postdilatation were performed in 92 and 49% of lesions, respectively. The difference between the diameter of the predilatation balloon and the reference vessel diameter was significantly associated with an increased risk for LOCE (odds ratio 4.84, 95% confidence interval: 1.91–12.7) with significant interaction with diabetes (*p* for interaction = 0.04), thus disfavouring predilatation with oversized balloons.

**Conclusion:**

The low LOCE rate (7.7%) over 5 years underscores the efficacy of PCI with EES. The use of ‘oversized’ balloons for predilatation was associated with an increased risk of LOCE by up to fivefold, a risk that was interestingly reduced in patients with diabetes mellitus.

**Supplementary Information:**

The online version of this article (10.1007/s12471-025-01937-4) contains supplementary material, which is available to authorized users.

## What’s new?

- This study of the AIDA trial enrolling patients undergoing percutaneous coronary intervention (PCI) investigated the impact of stent implantation steps (i.e. predilatation, stent sizing and postdilatation; also referred to as PSP) in the drug-eluting stent (DES) arm.
- Our observations reveal that, in non-complex lesions, utilisation of oversized balloons during predilatation significantly increases the risk of lesion-oriented adverse events in PCI with DES. This risk is lower in diabetes, suggesting a role of plaque composition. The impact of stent implantation steps in more complex lesions remains to be determined.
- The refinement in implantation strategies, requiring minimal additional effort, presents an opportunity to further improve our already high-quality outcomes (notably low stent thrombosis rates) in daily PCI practice with DES.

## Introduction

Long-term results of percutaneous coronary intervention (PCI) have improved significantly due to advancements in devices, guidance by intravascular imaging and improvements in PCI techniques with cutting/scoring balloons or rotational atherectomy. These include thinner struts, improvements in the biocompatibility of polymer coating, and the profile of the eluted drug-facilitated improvement in short-and long-term outcomes of PCI with a subsequent reduction in the need for reinterventions. Nevertheless, suboptimal device implantation with intracoronary angiography may still contribute to increased rates of adverse events after PCI with drug-eluting stents (DES) [1, 2].

In post hoc analyses, meticulous angiography-guided implantation strategies (predilatation, scaffold sizing and postdilatation; also referred to as PSP) seemed to mitigate procedure-related adverse events in PCI with bioresorbable scaffolds (BVS), such as early stent thrombosis (ST, < 30 days) and target lesion revascularisation (TLR) [3–7]. Importantly, data from randomised controlled trials on this topic are lacking, and the degree of impact of applying these strategies to PCI with DES devices is not fully elucidated.

The final and 5‑year patient-related clinical outcomes of the AIDA trial, comparing PCI with BVS to everolimus-eluting stents (EES), have been published previously [8]. In this subanalysis, we present the final 5‑year lesion-oriented outcomes of the patients treated with DES, whereby we evaluated the impact of lesion preparation and stent optimisation on long-term outcomes using quantitative coronary angiography (QCA) in lesions treated with EES (Xience Family; Abbott, Veenendaal, The Netherlands).

## Methods

### AIDA trial

The AIDA trial was an investigator-initiated, multicentre, all-comer, randomised controlled trial enrolling patients with obstructive coronary artery disease (CAD) eligible for PCI across five sites in the Netherlands. The study design was approved by the institutional research ethical committee board at the Academic Medical Centre in Amsterdam. Enrolled patients were randomised between PCI with BVS or EES in a 1:1 ratio, and all patients received periprocedural antithrombotic therapy according to standard guideline-recommended regimes. In patients with acute coronary syndrome, dual antiplatelet therapy (DAPT), preferably with ticagrelor or prasugrel, was recommended for at least 1 year after the index procedure in both treatment arms.

Prespecified follow-ups were conducted at 1 month, 6 months and annually thereafter, up to 5 years post-index PCI. Patients’ clinical status and cardiac pharmacotherapy were assessed during these prespecified follow-up visits. An independent data and safety monitoring board ensured the safety of the participants as cumulative safety data were reviewed at regular intervals. Adverse events were adjudicated by an independent clinical event committee (Cardialysis B.V., Rotterdam, The Netherlands) according to the definitions of the Academic Research Consortium and the fourth universal definition of myocardial infarction [9, 10].

### Lesion sample and QCA analysis

Procedural coronary angiograms of patients eligible for QCA with at least one implanted EES were included in this retrospective study. QCA analyses were performed using validated offline software (Cardiovascular Angiography Analysis System, version 5.11; Pie Medical Imaging, Maastricht, The Netherlands) by seven experienced QCA analysts supervised by one QCA expert (Y.O.), all blinded for events.

QCA analyses were performed using single angiographic views. In cases where multiple projections were available, the projection displaying the most severe stenosis was selected. Vessel diameters at various locations around the lesion were quantified by QCA, including minimum lumen diameter (MLD), lesion length, maximum vessel diameters (D_max_) proximal and distal to the stented segment, and the reference vessel diameter (RVD), defined as the interpolated average from the proximal RVD and distal RVD.

### Implantation optimisation, primary and secondary outcomes

**Fig. 1:**
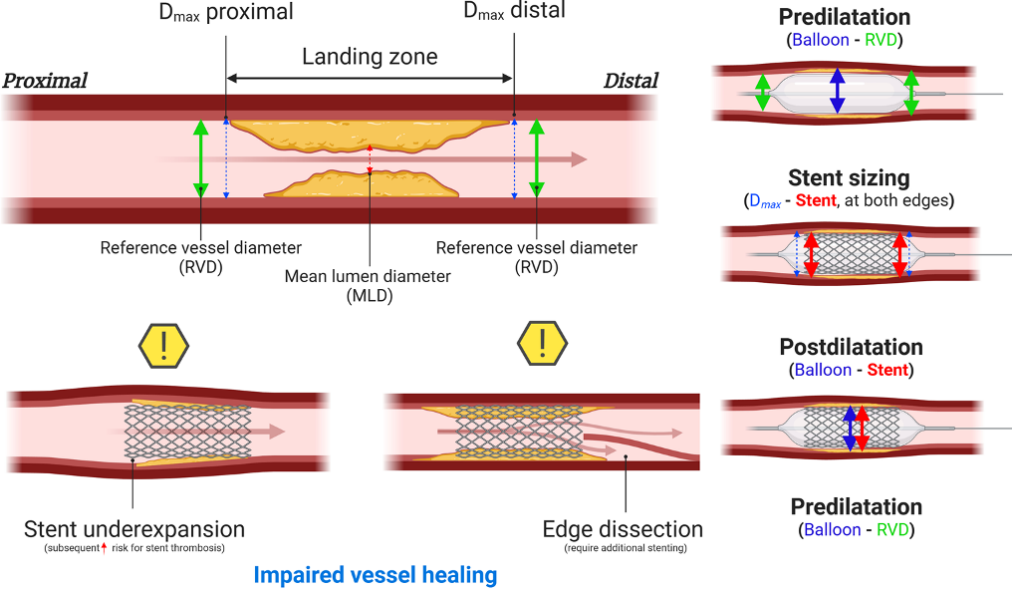
Illustrated overview of predilatation, stent implantation and postdilatation procedures (created with BioRender.com). *D*_*max*_ maximum vessel diameter

The cornerstone components of lesion preparation and stent implantation (i.e. predilatation, stent implantation and postdilatation) were analysed as continuous variables based on the relationships between vessel diameters at various locations around the treated lesion (Fig. 1). For predilatation, analysis focused on the difference in diameter between predilatation balloon and RVD. Stent implantation was evaluated by comparing the D_max_ to the stent diameter at both stent edges. A D_max_ minus stent diameter difference of less than 0.5 mm, indicating minor oversizing or undersizing, was considered to qualify as ‘D_max_ matched stent sizing’. Postdilatation analyses involved assessing the residual MLD in relation to the difference between postdilatation balloon diameter and stent diameter. Importantly, intravascular imaging by intravascular ultrasound (IVUS) or optical coherence tomography (OCT) was not mandated per protocol.

The impact of these lesion preparation and optimisation strategies on the occurrence of lesion-oriented composite events (LOCE) up to 5 years post-index PCI was the primary outcome of this study. LOCE were defined as a composite of TLR, target vessel myocardial infarction (TV-MI) and ST at the lesion level.

### Statistical analysis

Categorical data were summarised with counts and percentages, continuous data with means ± SD. Pearson’s chi-squared and Student’s *t*-tests were applied for categorical and continuous variable comparison, respectively. Event rates were estimated using the Kaplan-Meier method. Logistic regression analyses assessed the impact on LOCE, with predictors identified via univariate logistic regression and refined multivariate models using the glmulti package, favouring Akaike’s IC over *R*^2^ to avoid overfitting. *p*-values < 0.05 were considered statistically significant. Analyses were conducted using the R Statistical language (version 4.3.1; R Core Team, 2023) on macOS Sonoma 14.1.1 with all packages detailed in the Electronic Supplementary Material.

## Results

### Study population and lesion characteristics

In the AIDA trial, 1845 patients with 2446 lesions were treated between 2013 and 2015. In total, 921 patients and 1212 lesions were treated with EES. A total of 1098 (90%) lesions suitable for QCA analyses were included in this study. The patients’ baseline characteristics are summarised in Table S1 (Electronic Supplementary Material). The median anatomical SYNTAX score was 11 (interquartile range (IQR): 7–17). More than half of the lesions (54.8%, *n* = 602) were treated in the context of acute coronary syndromes. A third of the lesions (29.2%, *n* = 321) were located in the right coronary artery, 476 (43.4%) in the left anterior descending (LAD) artery and 291 (26.5%) in the left circumflex artery. The majority of lesions were treated with a single stent (91%, *n* = 995), 819 (74.6%) were ≤ 20 mm in length, 795 (72.4%) lesions had a diameter > 2.75 mm, and 25 (2.3%) lesions underwent rotablation (Tab. 1).

**Table 1:** Lesion and procedural characteristics

Characteristic	Everolimus-eluting stent (*n* = 1098)	Characteristic	Everolimus-eluting stent (*n* = 1098)
*Target vessel, n (%)*		Predilatation performed, *n* (%)	1013 (92%)
RCA	321 (29%)	*Predilatation balloon diameter (mm), n (%)*	
LM	6 (0.5%)	2	116 (11%)
LAD	476 (43%)	2.25	1 (< 0.1%)
LCX	291 (27%)	2.5	543 (50%)
IM/AL	0 (0%)	2.75	5 (0.5%)
Arterial bypass graft	0 (0%)	3	285 (26%)
Venous bypass graft	4 (0.4%)	3.25	2 (0.2%)
*Number of stents per lesion, n (%)*		3.5	56 (5.1%)
1	995 (91%)	3.75	0 (0%)
2	98 (8.9%)	4	3 (0.3%)
3	5 (0.5%)	Not applicable/not performed	85 (7.8%)
Total occlusion, *n* (%)	156 (14%)	Predilatation balloon length (mm), median (IQR)	15.00 (15.00, 20.00)
Ostial lesion, *n* (%)	62 (5.7%)	Predilatation balloon pressure (atm), median (IQR)	10.00 (10.00, 12.00)
Bifurcation lesion, *n* (%)	63 (5.7%)	Total stent length per lesion, Median (IQR)	18 (15, 28)
*Calcified lesion, n (%)*		Mean stent pressure per lesion (atm), median (IQR)	14.00 (12.00, 15.00)
None	265 (24%)	Postdilatation performed, *n* (%)	538 (49%)
Mild	535 (49%)	Postdilatation performed with NC, *n* (%)	457 (42%)
Moderate	209 (19%)	*Postdilatation balloon diameter (mm), n (%)*	
Severe	89 (8.1%)	1.5	1 (0.2%)
Rotablation performed, *n* (%)	25 (2.3%)	2	4 (0.7%)
*Vessel diameter, n (%)*		2.5	51 (9.5%)
≤ 2.75 mm	301 (27%)	2.75	9 (1.7%)
> 2.75 mm	795 (73%)	3	173 (32%)
*Lesion length, n (%)*		3.25	20 (3.7%)
≤ 20 mm	819 (75%)	3.5	196 (36%)
> 20 mm	279 (25%)	3.75	11 (2.0%)
*AHA lesion classification, n (%)*		4	58 (11%)
A	114 (10%)	4.5	12 (2.2%)
B1	433 (40%)	5	2 (0.4%)
B2	380 (35%)	Postdilatation balloon length (mm), median (IQR)	15.0 (12.0, 15.0)
C	169 (15%)	Postdilatation balloon pressure (atm), median (IQR)	16.0 (14.0, 18.0)

*RCA* right coronary artery, *LM* left main artery, *LAD* left anterior descending artery, *LCX* left circumflex artery, *IM/AL* intermediate/anterolateral artery, *IQR* interquartile range, *NC* non-compliant balloon, *AHA* American Heart Association

### LOCE, lesion preparation and implantation steps

LOCE occurred in 84 (7.7%) lesions, mainly driven by TLR (5.7%, *n* = 63) and by TV-MI (4.2%, *n* = 46), predominantly occurring within the first 3 years after index PCI. ST occurred in 9 (0.8%) lesions (Fig. S1, Table S2; Electronic Supplementary Material). Predilatation was performed in 1013 (92.3%) lesions, D_max_ matched stent sizing in 471 (42.8%), and lesion postdilatation in 538 (49%) (Fig. S2, Electronic Supplementary Material). Predilatation was performed with a median predilatation balloon length of 15 mm (IQR: 15–20 mm) at median balloon pressures of 10 atm (IQR: 10–12 atm). The overall median stent length per lesion was 18 mm (IQR: 15–28 mm), applied at a median pressure of 14 atm (IQR: 12–15 atm). Postdilatation balloons had a median length of 15 mm (IQR: 12–15 mm) with median inflation pressures of 16 atm (IQR: 14–18 atm) (Tab. 1).

The Welch two-sample *t*-test shows that for lesions with LOCE, predilatation balloon diameters exceeded RVD by +0.13 mm (95% confidence interval (CI): 0.04, 0.22), whereas postdilatation balloons were smaller than stent size by −0.10 mm (95% CI: −0.19, −0.01) (Tab. 2).

**Table 2:** Comparative analyses of lesion preparation and stent optimisation components between lesions with versus without LOCE

Characteristic	N	All lesions, (N = 1098)^a^	LOCE, (*n* = 84)	No LOCE, (*n* = 1014)	Difference^b^	*p*‑value^b^	95% CI^b^
Predilatation, *n* (%)	1098	1013 (92%)	78 (93%)	935 (92%)	0.65%	> 0.9	
Difference in diameter between predilatation balloon and vessel (balloon-vessel diameter), mean ± SD	988	−0.39 ± 0.38	−0.27 ± 0.37	−0.40 ± 0.38	0.13	0.005	0.04, 0.22
Difference in proximal D_max_ and device diameter (D_max_—max. stent diameter), mean ± SD	1098	−0.22 ± 0.57	−0.24 ± 0.46	−0.22 ± 0.57	−0.02	0.7	−0.12, 0.09
Difference in distal D_max_ and device diameter (D_max_—max. stent diameter), mean ± SD	1098	−0.32 ± 0.52	−0.35 ± 0.49	−0.32 ± 0.52	−0.03	0.5	−0.14, 0.08
Difference in RVD and MLD post-stent, mean ± SD	1071	0.68 ± 0.34	0.62 ± 0.30	0.68 ± 0.34	−0.06	0.079	−0.13, 0.01
Postdilatation, *n* (%)	1098	538 (49%)	39 (46%)	499 (49%)	−2.8%	0.9	
Difference in diameters of postdilatation balloon and device (balloon-stent diameter), mean ± SD	537	0.16 ± 0.33	0.07 ± 0.27	0.17 ± 0.34	−0.10	0.037	−0.19, −0.01

*LOCE* lesion-oriented composite events, *CI* confidence interval, *D*_*max*_ maximum vessel diameter, *RVD* reference vessel diameter, *MLD* minimum lumen diameter

^a^*n* (%); mean ± SD

^b^Three-sample test for equality of proportions without continuity correction; Welch two-sample *t*-test

**Table 3:** Multivariate logistic regression analyses

Predictor	*n*	OR	95% CI	*p*‑value
*Target vessel*				
RCA	293	–	–	
LM	4	0.00		> 0.9
LAD	430	2.13	1.17, 4.06	0.017
LCX	256	1.06	0.50, 2.22	0.9
Venous bypass graft	4	3.90	0.18, 34.7	0.3
Prior CAD	290	1.62	0.85, 3.03	0.13
Rotablation performed	24	0.00		> 0.9
Predilatation | predilatation balloon—RVD	987	4.84	1.91, 12.7	0.001
Predilatation | predilatation balloon—RVD and prior CAD interaction	290	0.36	0.09, 1.36	0.13
Predilatation | predilatation balloon—RVD and diabetes interaction	987	0.30	0.10, 0.99	0.040
Postdilatation		0.72	0.43, 1.20	0.2

*OR* odds ratio, *CI* confidence interval, *RCA* right coronary artery, *LM* left main artery, *LAD* left anterior descending artery, *LCX* left circumflex artery, *CAD* coronary artery disease, *RVD* reference vessel diameter

### Lesion preparation, stent optimisation and impact on LOCE

**Fig. 2:**
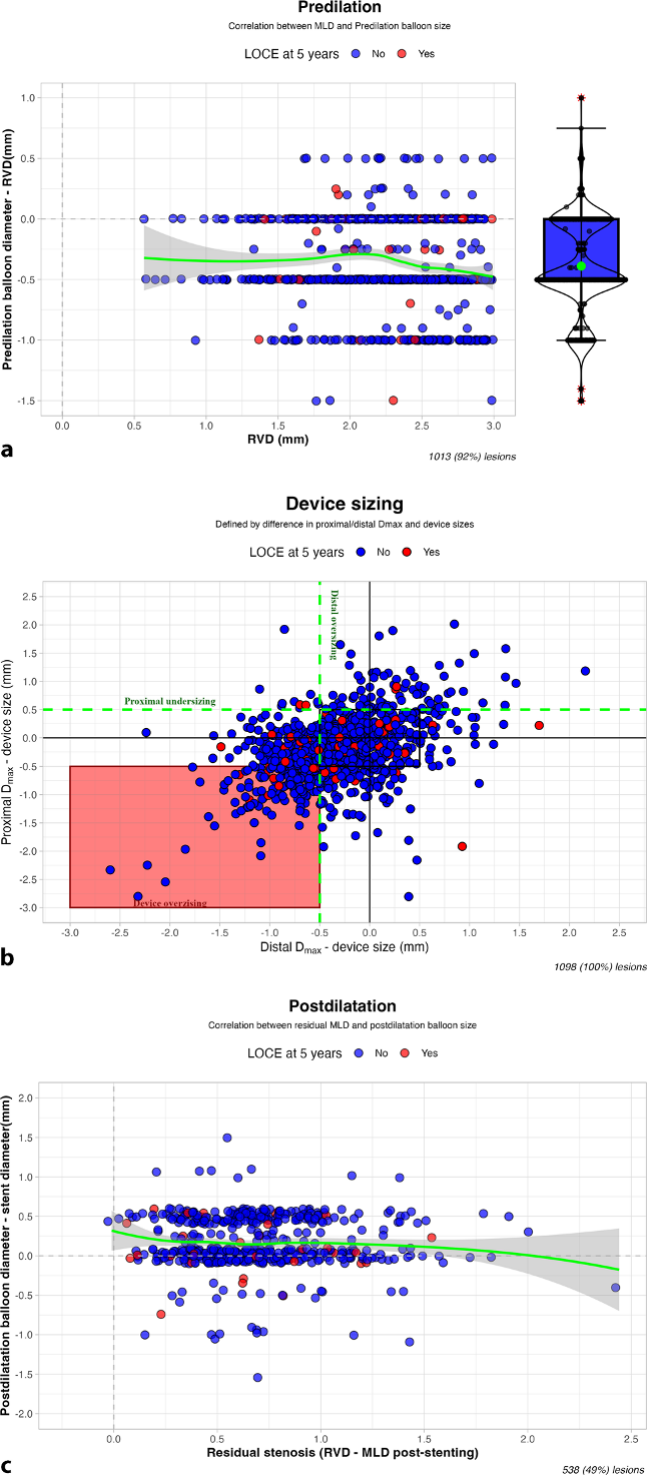
a**–c** In-depth comparison of lesion preparation and stent optimisation techniques. *MLD* minimum lumen diameter, *RVD* reference vessel diameter, *LOCE* lesion-oriented composite events

The majority of predilatated lesions (75%) were predilatated with balloons equal to or 0.5 mm smaller than the RVD (Fig. 2a). Subsequent stent sizing frequently matched the proximal and distal D_max_ in 42.9% of lesions, with instances of overall device oversizing at 17.7% and distal oversizing at 17.2% (Fig. 2b; Fig. S2, Electronic Supplementary Material). In a substantial number of lesions, device implantation was conducted with oversizing of the device (17.7% oversizing), 17.2% isolated oversizing at the distal stent edge. Figure 2c illustrates that postdilatation was most frequently performed with balloons with equally sized or larger diameters relative to the stent diameter, irrespective of the difference in RVD and residual MLD.

Diabetes mellitus, prior CAD, target vessel, rotablation and the difference in diameter between predilatation balloon and RVD, as well as between postdilatation balloon and stent diameter, emerged as potential predictors of LOCE in univariate logistic regression analyses (Table S3, Electronic Supplementary Material).

**Fig. 3:**
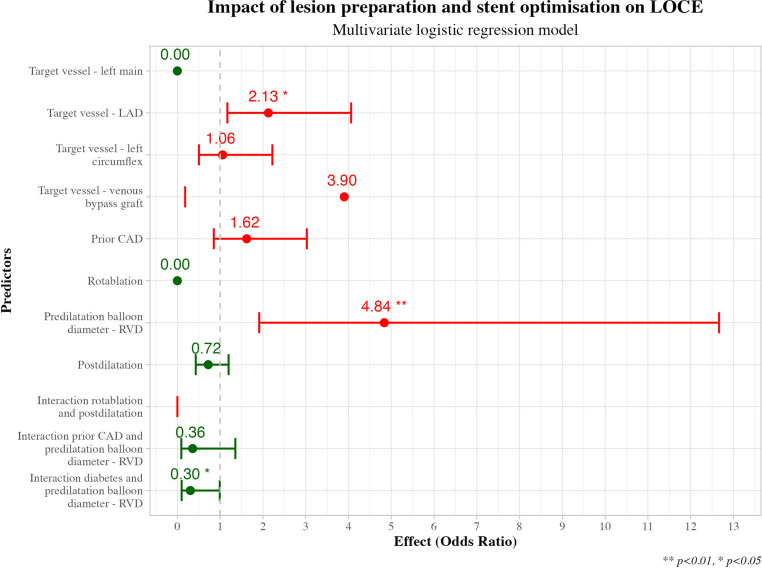
Logistic regression analysis of predilatation and postdilatation balloon sizing and stent sizing on the probability of lesion-oriented composite events (*LOCE*). *LAD* left anterior descending artery, *CAD* coronary artery disease, *RVD* reference vessel diameter

A total of 65,314 multivariate logistic regression models were identified. The best model was fitted to predict 5‑year LOCE, including predictors diabetes, target vessel, rotablation, previous CAD, postdilatation and differences between the diameter of the predilatation balloon and the RVD. The model results are visualised in Tab. 3 and Fig. 3. With the LAD as target vessel, there is a statistically significant association with LOCE with an odds ratio (OR) of 2.13 (95% CI: 1.17, 4.06, *p* = 0.017). The difference between the diameter of the predilatation balloon and the RVD is strongly associated with LOCE (OR 4.84, 95% CI: 1.91–12.7, per 0.5 mm increase). Furthermore, this association is reversed in the presence of diabetes mellitus (OR 0.30, 95% CI: 0.10–0.99, per 0.5 mm increase). We fitted a multivariate linear model to assess factors associated with predilatation balloon sizing, revealing that smaller vessel diameter correlates with a larger balloon-to-RVD ratio, especially in chronic total occlusions (adjusted *R*^2^ = 0.02, *p* < 0.001; see Electronic Supplementary Material).

## Discussion

The main findings of this post hoc QCA subanalysis of the AIDA trial, investigating the impact of lesion preparation and stent optimisation strategies on long-term LOCE with EES in non-complex coronary anatomy (SYNTAX 11) are:

1. Lesions predilatated with oversized balloons relative to the RVD were more likely to experience LOCE. The risk increased up to fivefold with an oversized predilatation balloon relative to the RVD and also more than doubled with the LAD as the target vessel. Interestingly, this risk increase is mitigated in the presence of diabetes mellitus.
2. Compared to non-LOCE lesions, predilatation was typically performed with larger balloons, while postdilatation was conducted with smaller balloons relative to the RVD and stent diameter, respectively, in lesions with LOCE. Smaller vessel diameters were linked to larger predilatation balloon-to-RVD ratios. The low variance suggests that other factors may influence the operators’ choices of balloon size.

Current observations are in line with clinical practice in PCI with current-generation DES [11]. A low LOCE rate of 7.7%, primarily driven by target vessel revascularisation and TV-MI with the majority of events occurring within the first 3 years post-PCI and the very low incidence of definite ST (0.8%), similar to previous reports using the same device, are reassuring observations reflecting good everyday PCI practice [12]. LOCE, driven by TLR in 74% of cases, persist even when the stent struts are anticipated to be fully covered by a new endothelial layer 1 year after initial device implantation, considerably reducing the risk of ST and eliminating the necessity for DAPT [13]. Lesion predilatation was common (92.3%), with 75% of these lesions predilatated with balloons of equal size to the RVD or up to 0.5 mm smaller. Stent sizing matched the D_max_ at both edges in 42.8% of lesions, and oversizing (17.7%) or isolated oversizing at the distal stent edge (17.2%) were not uncommon. Nearly half of lesions (49%) were postdilatated. Predilatation and postdilatation were conducted using balloons with a median length of 15 mm at 10 atm and 16 atm, respectively, while the median stent length was 18 mm, implanted at 14 atm. Lesions predilatated with oversized balloons relative to the RVD were more likely to experience LOCE. Interestingly, this increase was partly mitigated in lesions of patients with diabetes mellitus. A different plaque composition likely contributes to different responses to barostressors applied during pre/postdilatation with a subsequent risk for LOCE [14].

The PSP technique for BVS involves predilatation using balloons oversized compared to the RVD and postdilatation with non-compliant balloons up to 0.5 mm larger than the scaffold’s nominal diameter at pressures ≥ 18 atm [15]. When applied to PCI with DES, the traditional PSP strategy elevates the risk of target lesion failure, primarily due to predilatation with oversized balloons [16, 17]. Further, optimal DES sizing (absolute difference between RVD and stent diameter ≤ 0.25 mm) may be a significant protective predictor [16]. Compared to our previous reports, we now adopted a more granular approach to analysing the components of meticulous DES implantation, treating them as continuous predictors rather than categorical variables [17, 18]. In complex CAD (e.g. left main artery, bifurcation lesions, diffuse lesions > 30 mm), IVUS-guided stent optimisation, especially post-dilatation, significantly reduces the risk of cardiac death and target vessel revascularisation [19, 20]. Reasonably, optimal implantation strategies may vary depending on disease complexity.

In lesions of low complexity, careful predilatation preventing intima barotrauma with a subsequent risk for (small) dissections and/or delayed vessel healing may enable effective plaque sealing with a properly sized DES, whereafter stent sizing and postdilatation have limited influence on LOCE risk. In drug-coated balloon treatment, small intima dissections are frequently captured with OCT, where the dissection volumes seem to correlate with late lumen loss [21]. Complex lesions in contrast, especially calcified lesions, may benefit from more aggressive predilatation to ensure optimal stent deployment in terms of strut apposition. Postdilatation using oversized non-compliant balloons at high pressures might improve clinical outcomes by ensuring optimal plaque sealing and stent strut apposition, though careful attention is needed to avoid iatrogenic vessel injury and edge dissection. Particularly in ST-elevation myocardial infarction patients and at distal stent edges in tapered vessels, where slow or no-flow complications can arise following aggressive dilation, caution is paramount [16]. Currently, Teeuwen et al. are performing a multicentre study on the impact of postdilatation in daily clinical practice using the NHR platform, which will provide us insights and further clarify this matter (personal communication).

Thus, improvements in outcomes can be made with relatively little effort during PCI procedures, but is angiography alone sufficient for optimal lesion selection, preparation and subsequent optimal device implantation?

As coronary CT angiography is very likely to become the primary screening tool for suspected CAD, and with computational fluid derived parameters (computed tomography-fractional flow reserve (FFR), angioFFR, quantitative flow ratio (QFR), Murray law-based QFR) derived from various coronary imaging modalities emerging at a rapid pace, our daily practice in treating CAD will very likely adapt to a more image-guided approach combining anatomical and physiological characteristics to improve outcomes [22, 23]. Therefore, results from ongoing clinical trials (P3 trial, P4 trial, FAVOR III Europe-Japan, PIONEER IV and MultiVessel Talent trials) incorporating these strategies in the PCI workflow are eagerly awaited [24–27].

### Limitations

This study’s limitations include its post hoc nature, potential underpowering, the non-mandatory (intracoronary) imaging or implantation strategies in the AIDA trial’s all-comer design, operator-dependent lesion preparation and stent optimisation may have introduced indication bias. Furthermore, the inability to use QCA in 10% of lesions may indicate more complex lesions are underrepresented. Importantly, image quality and angulations were not predefined by protocol. This high exclusion rate is largely explainable, as QCA requires high-quality imaging, correctly angled with sufficient contrast to accurately assess luminal contours for the assessment of the three device-implantation steps.

## Conclusion

In PCI with DES for lesions of low complexity (median SYNTAX of 11), predilatation with oversized balloons was associated with an up to fivefold increase in LOCE risk, which, interestingly, is partly mitigated in the presence of diabetes mellitus.

## Supplementary Information

The Supplementary Information includes several figures and tables that expand on the main results and methods. Figure S1 shows cumulative event curves for LOCE, TLR, TV-MI, and ST, while Figure S2 provides barplots illustrating the frequencies of different device implantation steps. Figure S3 details diameter measurements across the stented segments. Figures S4 and S5 graphically present a multivariate linear model used to predict predilation balloon sizing, and Figure S6 shows a density plot comparing lesion lengths based on balloon oversizing. Tables S1, S2, and S3 contain baseline characteristics, event data, and univariate logistic regression analyses, respectively. Finally, a list of R packages used in the analyses is also provided.
