# Supplementary material for: Impact of lesion preparation and stent optimisation on lesion-oriented events in PCI with drug-eluting stents: 5-year results from the AIDA trial
Source: Neth Heart J. 2025 Mar 6;33(4):130–7. doi: 10.1007/s12471-025-01937-4 (PMC11953485; doi:10.1007/s12471-025-01937-4)
Supplement: Supplementary file 1 — The Supplementary Information includes several figures and tables that expand on the main results and methods. Figure S1 shows cumulative event curves for LOCE, TLR, TV-MI, and ST, while Figure S2 provides barplots illustrating the frequencies of different device implantation steps. Figure S3 details diameter measurements across the stented segments. Figures S4 and S5 graphically present a multivariate linear model used to predict predilation balloon sizing, and Figure S6 shows a density plot comparing lesion lengths based on balloon oversizing. Tables S1, S2, and S3 contain baseline characteristics, event data, and univariate logistic regression analyses, respectively. Finally, a list of R packages used in the analyses is also provided. [file 12471_2025_1937_MOESM1_ESM.docx]

Supplementary Material

**Supplementary material**

1. Figure S1. Cumulative event curves of LOCE, TLR, TV-MI and ST. 2
2. Figure S2. Barplots To Illustrate the Frequencies at which the device implantation steps are performed.
3. Figure S3. Diameter measurements each multiple locations around the stented segments
4. Figure S4+5. Graphical representation of a Multivariate Linear Model for Predicting Predilation Balloon Sizing
5. Figure S6. Density plot of lension length by predilation with oversized balloon.
6. Table S1. Baseline characteristics
7. Table S2. Events
8. Table S3. Univariate logistic regression analyses
9. R packages used.

## Figure S1

**
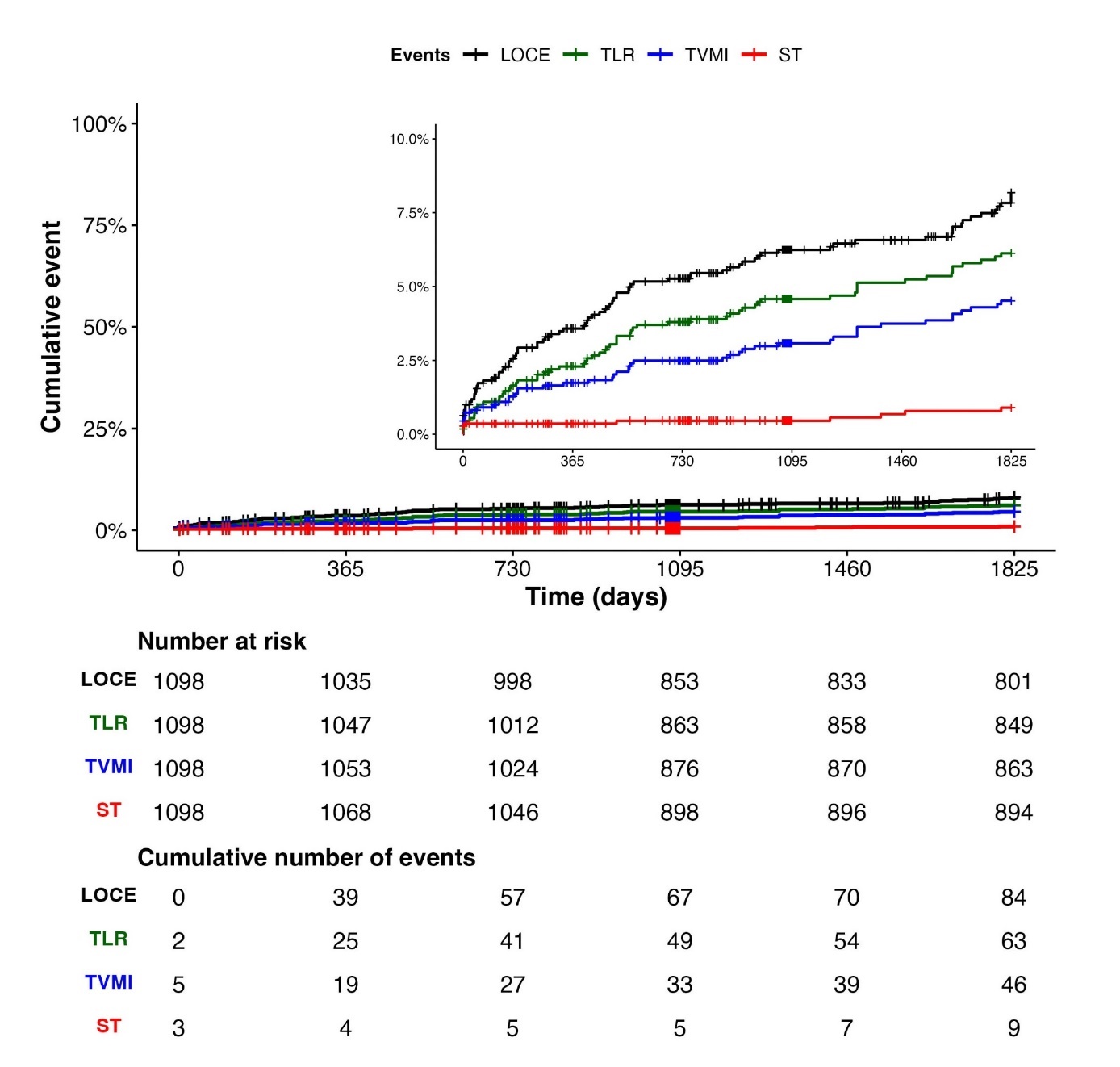
**

## Figure S2

##
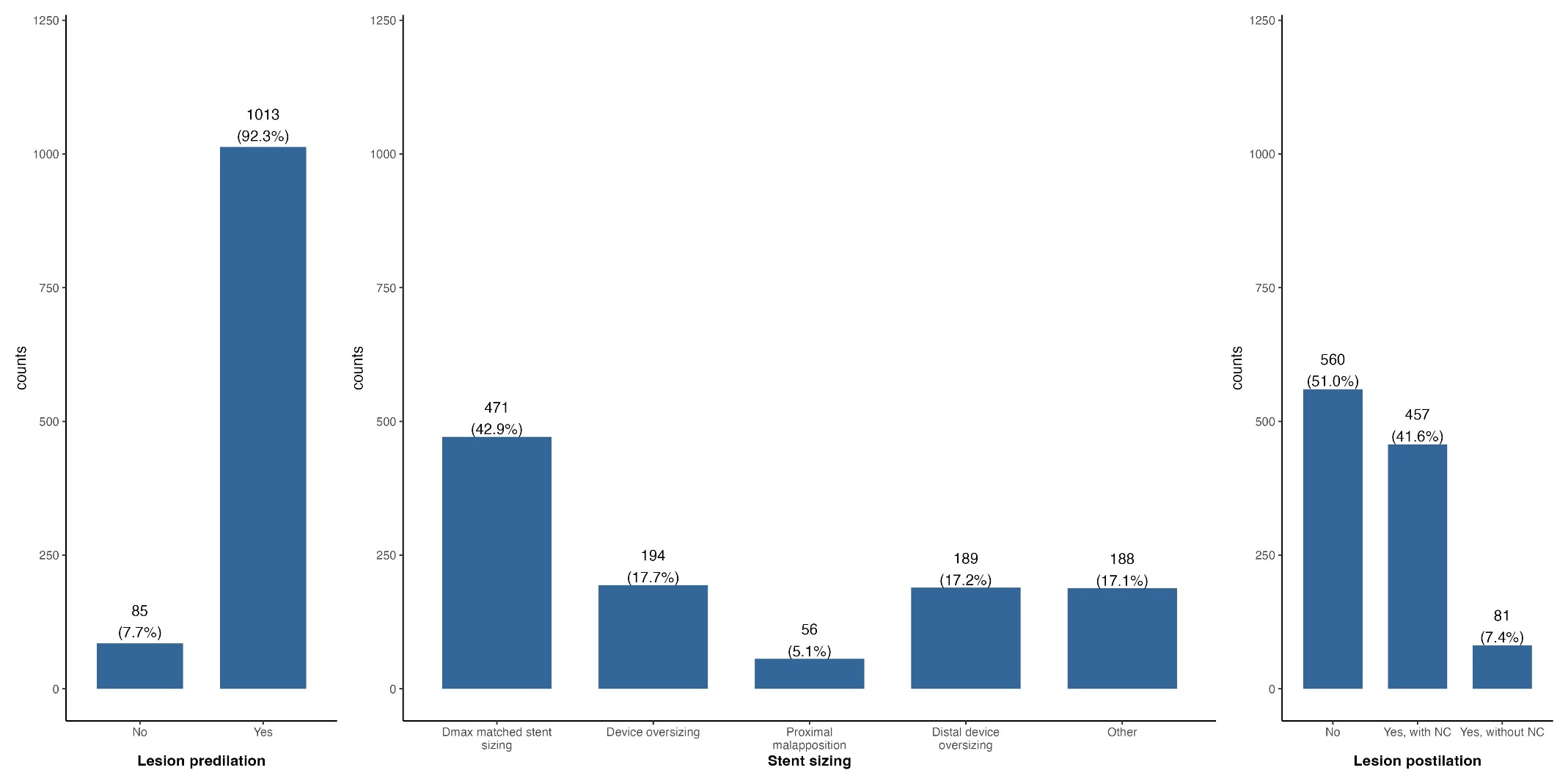


## Figure S3


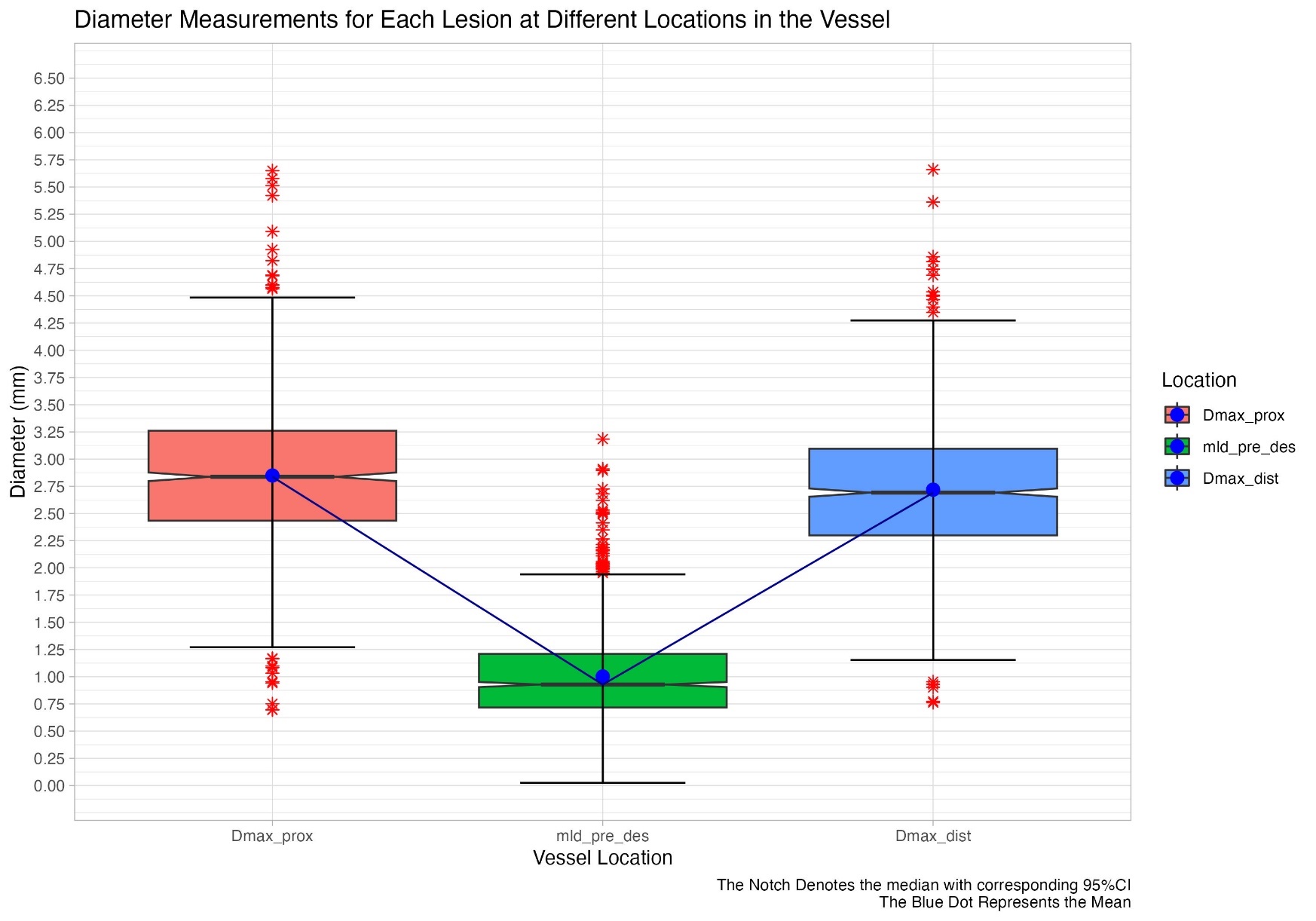


## Figure S4

| **Table. Multivariate Linear Model to Predict Predilation Balloon Sizing** | | | |
| --- | --- | --- | --- |
|  | **Ratio between Predilation Balloon and RVD** | | |
| *Predictors* | *Estimates* | *CI* | *p* |
| (Intercept) | 0.94 | 0.91 – 0.98 | **<0.001** |
| RVD pre-stent implantation (in mm) | -0.02 | -0.04 – -0.01 | **<0.001** |
| RVD pre-stent implantation in Total Occlusion | -0.02 | -0.03 – -0.01 | **<0.001** |
| R^2^ / R^2^ adjusted | 0.025 / 0.023 | | |


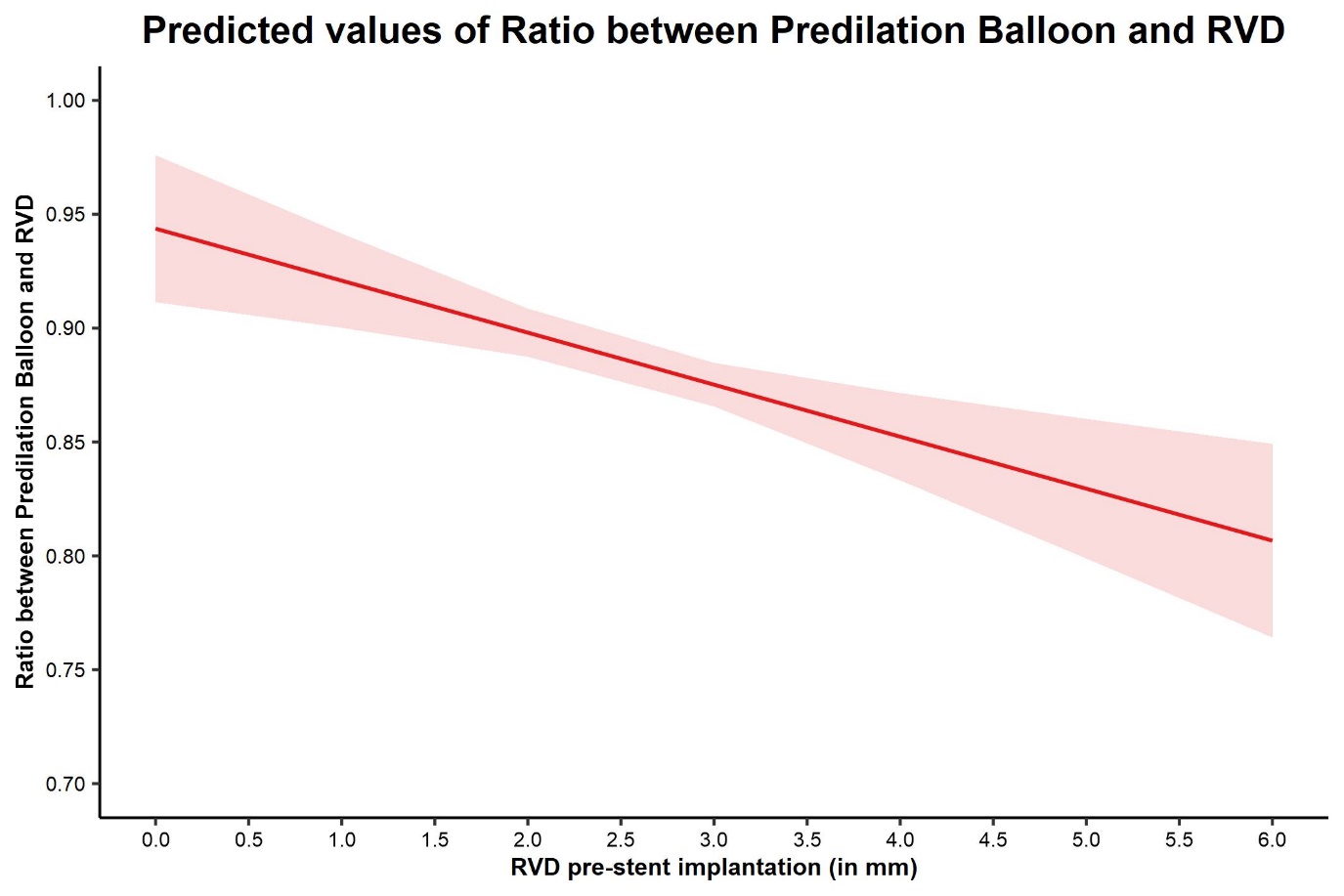


The model suggests that in smaller vessels, the predilation balloon is more oversized relative to the reference vessel diameter. The presence of a total occlusion amplifies the tendency for the predilation balloon to be relatively larger suggesting operators may deliberately choose larger balloons relative to vessel size to navigate and open these challenging lesions effectively.

## Figure S5


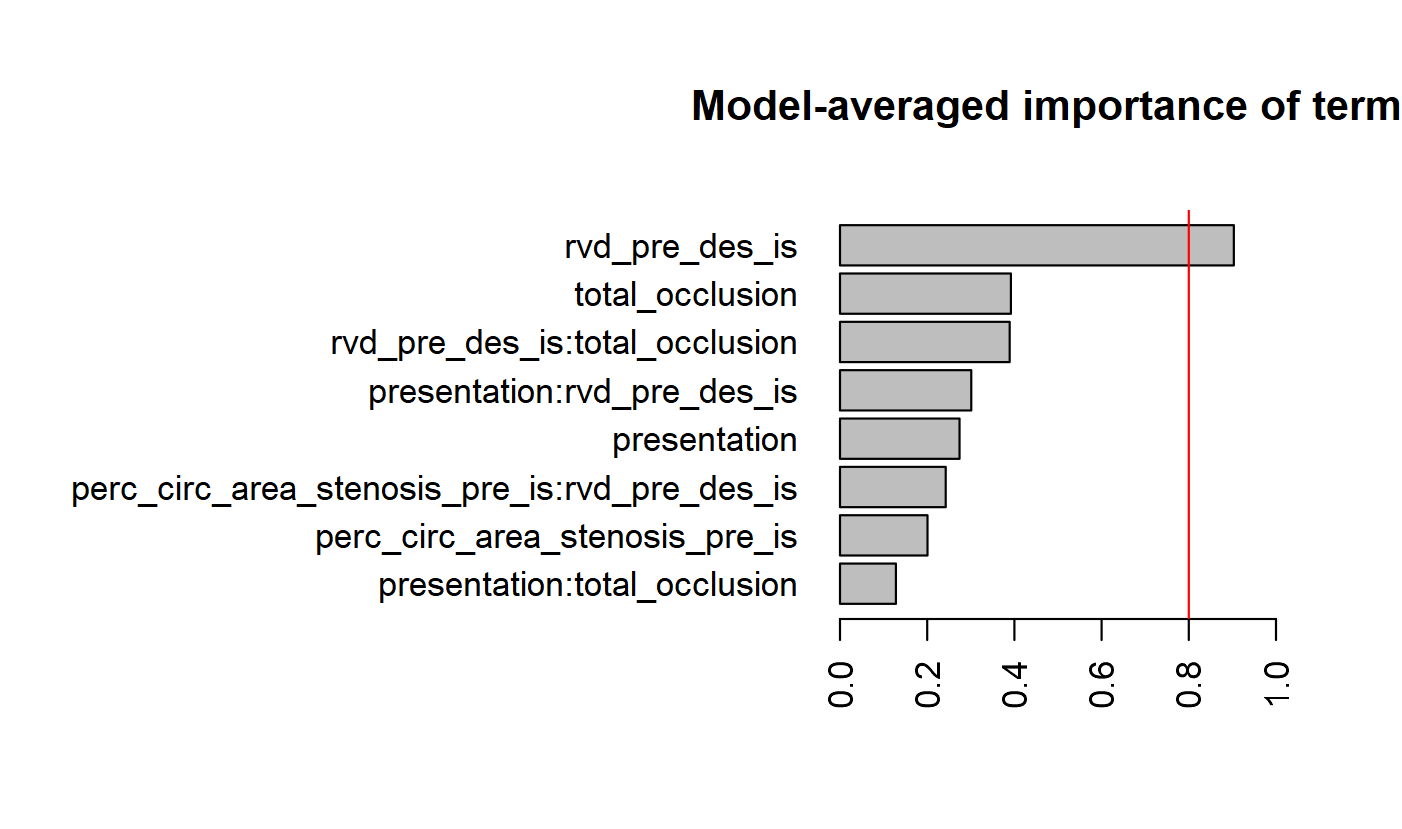


In the 5000 potential models, the RVD pre-stent implantation (rvd_pre_des_is) was by far the most important term to predict the Ratio between the diameters of the Predilation Balloon and RVD (which represents predilation balloon sizing relative to the reference diameter)
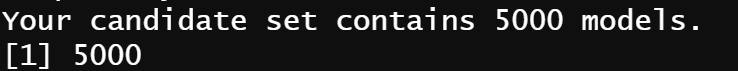
.

## Figure S6.


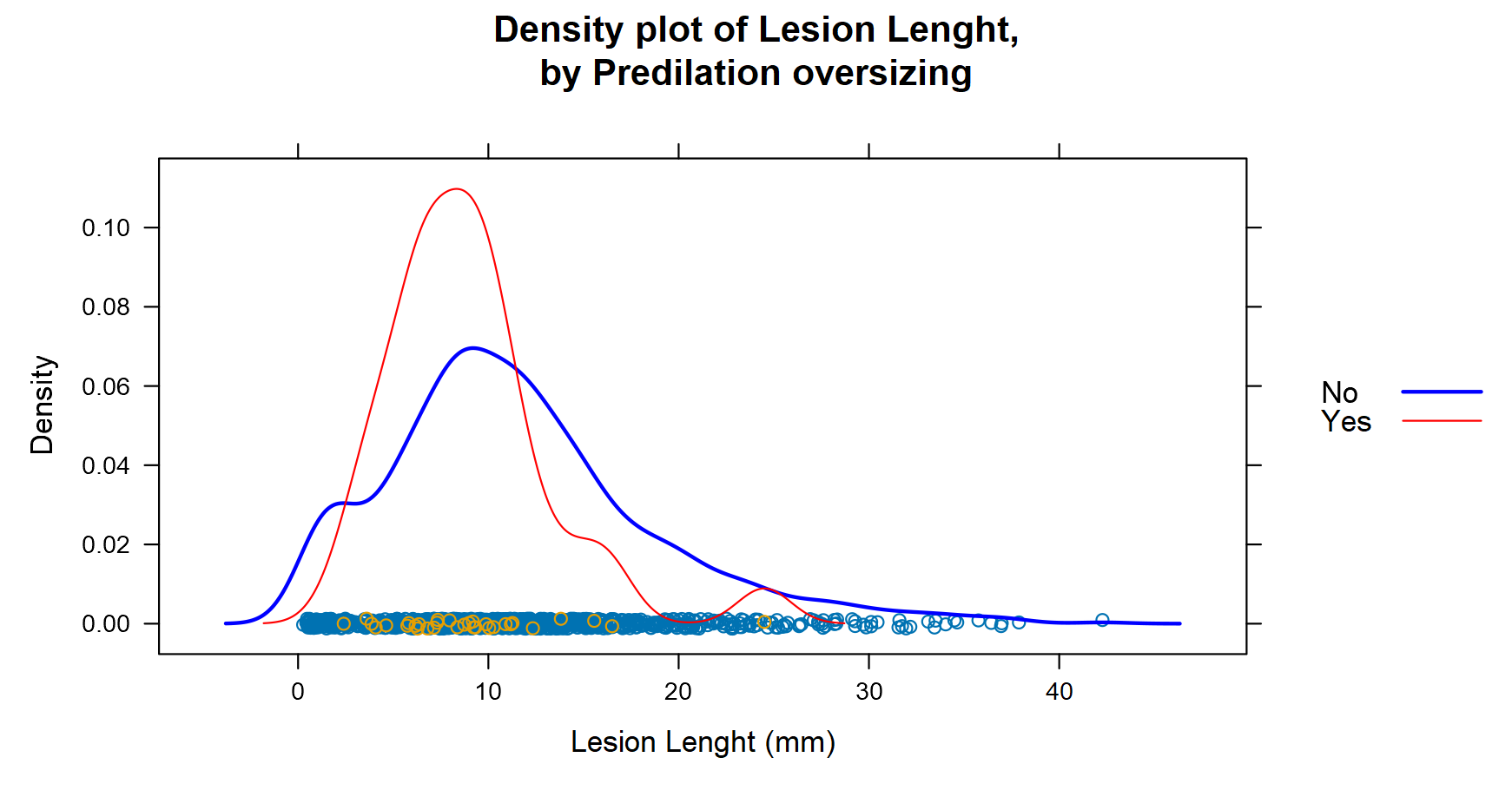


This density plot compares lesion lengths based on predilation balloon sizing. The red curve represents lesions predilated with oversized balloons, and the blue curve represents those predilated with undersized or appropriately sized balloons. The plot displays the distribution of lesion lengths as densities; multiplying these densities by 100 converts them into percentage values of the total observations for each category. Overall the distribution of the lesion lengths were: 1st Quartile = 6.9mm , Median = 10.4mm Mean = 11.4mm, 3rd Quartile = 14.5.

## Table S1. Baseline characteristics

|  | Patient level | Lesion level |
| --- | --- | --- |
| **Characteristic** | **N = 921** | **N = 1,098** |
| **Presentation at Index, n (%)** |  |  |
| CCS | 417 (45%) | 496 (45%) |
| NSTEMI | 192 (21%) | 237 (22%) |
| STEMI | 225 (24%) | 251 (23%) |
| Unstable Angina | 87 (9.4%) | 114 (10%) |
| **Age (years), Mean (SD)** | 64 (11) |  |
| **Sex at birth, n (%)** |  |  |
| Female | 221 (24%) |  |
| Male | 700 (76%) |  |
| **Smoking status, n (%)** |  |  |
| Current | 273 (32%) |  |
| Never | 279 (32%) |  |
| Previous | 309 (36%) |  |
| Unknown | 60 |  |
| **Family history of CAD, n (%)** | 469 (53%) |  |
| **Diabetes Mellitus, n (%)** | 153 (17%) |  |
| **Dyslipidemia, n (%)** | 350 (38%) |  |
| **Hypertension, n (%)** | 464 (50%) |  |
| **Previous CAD, n (%)** | 270 (29%) |  |
| **Previous MI, n (%)** | 172 (19%) |  |
| **Previous PCI, n (%)** | 184 (20%) |  |
| **Previous CABG, n (%)** | 46 (5.0%) |  |
|  |  |  |
| **Chronic kidney disease (CKD), n (%)** | 91 (9.9%) |  |
| **SYNTAX-I score (Anatomical), Median (IQR)** | 11 (7, 17) |  |
| **Location of lesion** |  |  |
| RCA |  | 321 (29%) |
| LM |  | 6 (0.5%) |
| LAD |  | 476 (43%) |
| CX |  | 291 (27%) |
| Venous bypass graft |  | 4 (0.4%) |
| **Lesion length category** |  |  |
| ≤20mm |  | 819 (75%) |
| >20mm |  | 279 (25%) |
| **Lesion size category** |  |  |
| ≤2.75mm |  | 301 (27%) |
| >2.75mm |  | 795 (73%) |
| **Amount of lesions** |  |  |
| 1 |  | 866 (81%) |
| 2 |  | 171 (16%) |
| 3 |  | 28 (2.6%) |
| 4 |  | 4 (0.4%) |

## Table S2. Events

|  | **Patient level** | **Lesion level** |
| --- | --- | --- |
| **Characteristic** | **N = 921** | **N = 1,098** |
| **All-cause death, n (%)** | 88 (9.6%) |  |
| **Cardiac death, n (%)** | 41 (4.5%) |  |
| **Myocardial infarction (MI), n (%)** | 62 (6.7%) |  |
| **Target Vessel Myocardial infarction (TV-MI), n (%)** | 44 (4.8%) | 46 (4.2%) |
| **Target Vessel Myocardial infarction, Index procedure related, n (%)** | 6 (0.7%) |  |
| **Definite Stent Thrombosis (ST), n (%)** | 9 (1.0%) | 9 (0.8%) |
| **Target Lesion Revascularization (TLR), n (%)** | 64 (6.9%) | 63 (5.7%) |
| **Lesion Oriented Composite Endpoint (LOCE)** |  | 84 (7.7%) |

## Table S3. Univariate logistic regression analyses

| **Predictor** | **N** | **OR**^1^ | **95% CI**^1^ | **p-value** |
| --- | --- | --- | --- | --- |
| **Age (years)** | 1,098 | 0.99 | 0.96, 1.01 | 0.2 |
| **Sex at birth** |  |  |  |  |
| *Male* | 832 | — | — |  |
| *Female* | 266 | 1.05 | 0.61, 1.72 | 0.9 |
| **BMI** | 1,092 | 1.02 | 0.97, 1.08 | 0.4 |
| **Family history of CAD** |  | 1.13 | 0.72, 1.81 | 0.6 |
| **Smoking status** |  |  |  |  |
| *Never* | 339 | — | — |  |
| *Current* | 314 | 1.23 | 0.70, 2.20 | 0.5 |
| *Previous* | 373 | 0.90 | 0.50, 1.63 | 0.7 |
| **Hypertension** |  | 1.32 | 0.85, 2.09 | 0.2 |
| **Dyslipidemia** |  | 0.79 | 0.49, 1.25 | 0.3 |
| **Diabetes Mellitus** |  | 1.65 | 0.96, 2.74 | 0.058 |
| **Diabetes treatment** |  |  |  |  |
| *Not Applicable* | 907 | — | — |  |
| *No* | 12 | 1.22 | 0.07, 6.41 | 0.9 |
| *Oral* | 123 | 1.86 | 0.99, 3.30 | 0.042 |
| *Insulin* | 53 | 1.40 | 0.47, 3.32 | 0.5 |
| **History of myocardial infarction** |  | 1.54 | 0.90, 2.56 | 0.10 |
| **History of PCI** |  | 2.46 | 1.51, 3.92 | <0.001 |
| **History of CABG** |  | 4.53 | 2.18, 8.86 | <0.001 |
| **Presentation at index** |  |  |  |  |
| *CCS* | 496 | — | — |  |
| *Unstable Angina* | 114 | 1.32 | 0.63, 2.60 | 0.4 |
| *NSTEMI* | 237 | 0.84 | 0.44, 1.53 | 0.6 |
| *STEMI* | 251 | 1.13 | 0.64, 1.96 | 0.7 |
| **Target Vessel** |  |  |  |  |
| *RCA* | 321 | — | — |  |
| *LM* | 6 | 0.00 |  | >0.9 |
| *LAD* | 476 | 1.89 | 1.10, 3.39 | 0.027 |
| *LCX* | 291 | 1.04 | 0.52, 2.07 | >0.9 |
| *Venous bypass graft* | 4 | 5.61 | 0.27, 46.4 | 0.14 |
| **ACC/AHA Lesion Type** |  |  |  |  |
| *A* | 114 | — | — |  |
| *B1* | 433 | 1.09 | 0.51, 2.61 | 0.8 |
| *B2* | 380 | 1.01 | 0.47, 2.45 | >0.9 |
| *C* | 169 | 1.39 | 0.59, 3.53 | 0.5 |
| **>1 stent** |  | 1.57 | 0.84, 2.77 | 0.13 |
| **Total occlusion** |  | 0.61 | 0.27, 1.22 | 0.2 |
| **Ostial lesion** |  | 1.31 | 0.49, 2.92 | 0.5 |
| **Bifurcation lesion** |  | 0.81 | 0.24, 2.03 | 0.7 |
| **Calcified lesion** |  |  |  |  |
| *None* | 265 | — | — |  |
| *Mild* | 535 | 0.91 | 0.51, 1.65 | 0.7 |
| *Moderate* | 209 | 1.52 | 0.80, 2.92 | 0.2 |
| *Severe* | 89 | 1.28 | 0.51, 2.94 | 0.6 |
| **Rotablation performed** |  | 4.02 | 1.43, 9.83 | 0.004 |
| **Vessel diameter (mm)** |  |  |  |  |
| *≤2.75mm* | 301 | — | — |  |
| *>2.75mm* | 795 | 0.70 | 0.44, 1.13 | 0.13 |
| **Lesion size (mm)** |  |  |  |  |
| *≤20mm* | 819 | — | — |  |
| *>20mm* | 279 | 1.35 | 0.82, 2.17 | 0.2 |
| **Predilatation** |  | 1.10 | 0.50, 2.90 | 0.8 |
| **Minimum lumen diameter (MLD) prior to stenting** | 1,098 | 0.77 | 0.43, 1.32 | 0.4 |
| **Difference in diameter predilatation balloon and vessel (balloon-vessel diameter)** | 988 | 2.50 | 1.33, 4.78 | 0.005 |
| **Difference in proximal D_max_ and device diameter (Dmax- Max stent diameter)** | 1,098 | 0.94 | 0.64, 1.40 | 0.8 |
| **Difference in distal D_max_ and device diameter (D_max_ - Max stent diameter)** | 1,098 | 0.88 | 0.57, 1.35 | 0.6 |
| **Reference vessel diameter post-procedure in total stented (prox - des - dist) segment** | 1,071 | 0.56 | 0.27, 1.12 | 0.11 |
| **Postdilatation** |  | 0.89 | 0.57, 1.40 | 0.6 |
| **Difference in diameters of postdilatation balloon and device (balloon-stent diameter)** | 537 | 0.45 | 0.19, 1.13 | 0.079 |
| ^1^OR = Odds Ratio, CI = Confidence Interval |  |  |  |  |

## R packages used

Analyses were conducted using the R Statistical language (version 4.3.1; R Core Team, 2023) on macOS Sonoma 14.1.1, using the packages gridExtra (version 2.3; Auguie B, 2017), lme4 (version 1.1.34; Bates D et al., 2015), Matrix (version 1.6.0; Bates D et al., 2023), maps (version 3.4.1.1; Becker OScbRA et al., 2023), effectsize (version 0.8.6; Ben-Shachar MS et al., 2020), broom.mixed (version 0.2.9.4; Bolker B, Robinson D, 2022), glmulti (version 1.0.8; Calcagno V, 2020), janitor (version 2.2.0; Firke S, 2023), effects (version 4.2.2; Fox J, Weisberg S, 2019), carData (version 3.0.5; Fox J et al., 2022), ggVennDiagram (version 1.2.3; Gao C, 2023), flextable (version 0.9.3; Gohel D, Skintzos P, 2023), cmprsk (version 2.2.11; Gray B, 2022), lubridate (version 1.9.2; Grolemund G, Wickham H, 2011), arsenal (version 3.6.3; Heinzen E et al., 2021), flexsurv (version 2.2.2; Jackson C, 2016), ggpubr (version 0.6.0; Kassambara A, 2023), survminer (version 0.4.9; Kassambara A et al., 2021), lmerTest (version 3.1.3; Kuznetsova A et al., 2017), sjmisc (version 2.8.9; Lüdecke D, 2018), sjPlot (version 2.8.15; Lüdecke D, 2023), performance (version 0.10.8; Lüdecke D et al., 2021), see (version 0.8.1; Lüdecke D et al., 2021), robustbase (version 0.99.0; Maechler M et al., 2023), report (version 0.5.7; Makowski D et al., 2023), leaps (version 3.1; Miller TLboFcbA, 2020), tibble (version 3.2.1; Müller K, Wickham H, 2023), writexl (version 1.4.2; Ooms J, 2023), ape (version 5.7.1; Paradis E, Schliep K, 2019), statsExpressions (version 1.5.1; Patil I, 2021), ggstatsplot (version 0.11.1; Patil I, 2021), nephro (version 1.3; Pattaro C, Fujii R, 2022), ggforce (version 0.4.1; Pedersen T, 2022), patchwork (version 1.1.2; Pedersen T, 2022), dynpred (version 0.1.2; Putter H, 2015), phytools (version 2.0.3; Revell L, 2012), broom (version 1.0.5; Robinson D et al., 2023), lattice (version 0.21.8; Sarkar D, 2008), latticeExtra (version 0.6.30; Sarkar D, Andrews F, 2022), openxlsx (version 4.2.5.2; Schauberger P, Walker A, 2023), plotly (version 4.10.3; Sievert C, 2020), gtsummary (version 1.7.2; Sjoberg D et al., 2021), survival (version 3.5.5; Therneau T, 2023), visdat (version 0.6.0; Tierney N, 2017), naniar (version 1.0.0; Tierney N, Cook D, 2023), rJava (version 1.0.10; Urbanek S, 2023), ggplot2 (version 3.4.4; Wickham H, 2016), stringr (version 1.5.0; Wickham H, 2022), forcats (version 1.0.0; Wickham H, 2023), tidyverse (version 2.0.0; Wickham H et al., 2019), readxl (version 1.4.3; Wickham H, Bryan J, 2023), dplyr (version 1.1.2; Wickham H et al., 2023), purrr (version 1.0.1; Wickham H, Henry L, 2023), readr (version 2.1.4; Wickham H et al., 2023), haven (version 2.5.3; Wickham H et al., 2023), tidyr (version 1.3.0; Wickham H et al., 2023), ggtext (version 0.1.2; Wilke C, Wiernik B, 2022), gridtext (version 0.1.5; Wilke C, Wiernik B, 2022) and knitr (version 1.43; Xie Y, 2023). References ---------- - Auguie B (2017). _gridExtra: Miscellaneous Functions for "Grid" Graphics_. R package version 2.3, <https://CRAN.R-project.org/package=gridExtra>. - Bates D, Mächler M, Bolker B, Walker S (2015). “Fitting Linear Mixed-Effects Models Using lme4.” _Journal of Statistical Software_, *67*(1), 1-48. doi:10.18637/jss.v067.i01 <https://doi.org/10.18637/jss.v067.i01>. - Bates D, Maechler M, Jagan M (2023). _Matrix: Sparse and Dense Matrix Classes and Methods_. R package version 1.6-0, <https://CRAN.R-project.org/package=Matrix>. - Becker OScbRA, Minka ARWRvbRBEbTP, team. ADFbtC (2023). _maps: Draw Geographical Maps_. R package version 3.4.1.1, <https://CRAN.R-project.org/package=maps>. - Ben-Shachar MS, Lüdecke D, Makowski D (2020). “effectsize: Estimation of Effect Size Indices and Standardized Parameters.” _Journal of Open Source Software_, *5*(56), 2815. doi:10.21105/joss.02815 <https://doi.org/10.21105/joss.02815>, <https://doi.org/10.21105/joss.02815>. - Bolker B, Robinson D (2022). _broom.mixed: Tidying Methods for Mixed Models_. R package version 0.2.9.4, <https://CRAN.R-project.org/package=broom.mixed>. - Calcagno V (2020). _glmulti: Model Selection and Multimodel Inference Made Easy_. R package version 1.0.8, <https://CRAN.R-project.org/package=glmulti>. - Firke S (2023). _janitor: Simple Tools for Examining and Cleaning Dirty Data_. R package version 2.2.0, <https://CRAN.R-project.org/package=janitor>. - Fox J, Weisberg S (2019). _An R Companion to Applied Regression_, 3rd edition. Sage, Thousand Oaks CA. <https://socialsciences.mcmaster.ca/jfox/Books/Companion/index.html>. Fox J, Weisberg S (2018). “Visualizing Fit and Lack of Fit in Complex Regression Models with Predictor Effect Plots and Partial Residuals.” _Journal of Statistical Software_, *87*(9), 1-27. doi:10.18637/jss.v087.i09 <https://doi.org/10.18637/jss.v087.i09>. Fox J (2003). “Effect Displays in R for Generalised Linear Models.” _Journal of Statistical Software_, *8*(15), 1-27. doi:10.18637/jss.v008.i15 <https://doi.org/10.18637/jss.v008.i15>. Fox J, Hong J (2009). “Effect Displays in R for Multinomial and Proportional-Odds Logit Models: Extensions to the effects Package.” _Journal of Statistical Software_, *32*(1), 1-24. doi:10.18637/jss.v032.i01 <https://doi.org/10.18637/jss.v032.i01>. - Fox J, Weisberg S, Price B (2022). _carData: Companion to Applied Regression Data Sets_. R package version 3.0-5, <https://CRAN.R-project.org/package=carData>. - Gao C (2023). _ggVennDiagram: A 'ggplot2' Implement of Venn Diagram_. R package version 1.2.3, <https://CRAN.R-project.org/package=ggVennDiagram>. - Gohel D, Skintzos P (2023). _flextable: Functions for Tabular Reporting_. R package version 0.9.3, <https://CRAN.R-project.org/package=flextable>. - Gray B (2022). _cmprsk: Subdistribution Analysis of Competing Risks_. R package version 2.2-11, <https://CRAN.R-project.org/package=cmprsk>. - Grolemund G, Wickham H (2011). “Dates and Times Made Easy with lubridate.” _Journal of Statistical Software_, *40*(3), 1-25. <https://www.jstatsoft.org/v40/i03/>. - Heinzen E, Sinnwell J, Atkinson E, Gunderson T, Dougherty G (2021). _arsenal: An Arsenal of 'R' Functions for Large-Scale Statistical Summaries_. R package version 3.6.3, <https://CRAN.R-project.org/package=arsenal>. - Jackson C (2016). “flexsurv: A Platform for Parametric Survival Modeling in R.” _Journal of Statistical Software_, *70*(8), 1-33. doi:10.18637/jss.v070.i08 <https://doi.org/10.18637/jss.v070.i08>. - Kassambara A (2023). _ggpubr: 'ggplot2' Based Publication Ready Plots_. R package version 0.6.0, <https://CRAN.R-project.org/package=ggpubr>. - Kassambara A, Kosinski M, Biecek P (2021). _survminer: Drawing Survival Curves using 'ggplot2'_. R package version 0.4.9, <https://CRAN.R-project.org/package=survminer>. - Kuznetsova A, Brockhoff PB, Christensen RHB (2017). “lmerTest Package: Tests in Linear Mixed Effects Models.” _Journal of Statistical Software_, *82*(13), 1-26. doi:10.18637/jss.v082.i13 <https://doi.org/10.18637/jss.v082.i13>. - Lüdecke D (2018). “sjmisc: Data and Variable Transformation Functions.” _Journal of Open Source Software_, *3*(26), 754. doi:10.21105/joss.00754 <https://doi.org/10.21105/joss.00754>. - Lüdecke D (2023). _sjPlot: Data Visualization for Statistics in Social Science_. R package version 2.8.15, <https://CRAN.R-project.org/package=sjPlot>. - Lüdecke D, Ben-Shachar M, Patil I, Waggoner P, Makowski D (2021). “performance: An R Package for Assessment, Comparison and Testing of Statistical Models.” _Journal of Open Source Software_, *6*(60), 3139. doi:10.21105/joss.03139 <https://doi.org/10.21105/joss.03139>. - Lüdecke D, Patil I, Ben-Shachar M, Wiernik B, Waggoner P, Makowski D (2021). “see: An R Package for Visualizing Statistical Models.” _Journal of Open Source Software_, *6*(64), 3393. doi:10.21105/joss.03393 <https://doi.org/10.21105/joss.03393>. - Maechler M, Rousseeuw P, Croux C, Todorov V, Ruckstuhl A, Salibian-Barrera M, Verbeke T, Koller M, Conceicao EL, Anna di Palma M (2023). _robustbase: Basic Robust Statistics_. R package version 0.99-0, <http://robustbase.r-forge.r-project.org/>. Todorov V, Filzmoser P (2009). “An Object-Oriented Framework for Robust Multivariate Analysis.” _Journal of Statistical Software_, *32*(3), 1-47. <https://www.jstatsoft.org/article/view/v032i03/>. - Makowski D, Lüdecke D, Patil I, Thériault R, Ben-Shachar M, Wiernik B (2023). “Automated Results Reporting as a Practical Tool to Improve Reproducibility and Methodological Best Practices Adoption.” _CRAN_. <https://easystats.github.io/report/>. - Miller TLboFcbA (2020). _leaps: Regression Subset Selection_. R package version 3.1, <https://CRAN.R-project.org/package=leaps>. - Müller K, Wickham H (2023). _tibble: Simple Data Frames_. R package version 3.2.1, <https://CRAN.R-project.org/package=tibble>. - Ooms J (2023). _writexl: Export Data Frames to Excel 'xlsx' Format_. R package version 1.4.2, <https://CRAN.R-project.org/package=writexl>. - Paradis E, Schliep K (2019). “ape 5.0: an environment for modern phylogenetics and evolutionary analyses in R.” _Bioinformatics_, *35*, 526-528. doi:10.1093/bioinformatics/bty633 <https://doi.org/10.1093/bioinformatics/bty633>. - Patil I (2021). “statsExpressions: R Package for Tidy Dataframes and Expressions with Statistical Details.” _Journal of Open Source Software_, *6*(61), 3236. doi:10.21105/joss.03236 <https://doi.org/10.21105/joss.03236>, <https://doi.org/10.21105/joss.03236>. - Patil I (2021). “Visualizations with statistical details: The 'ggstatsplot' approach.” _Journal of Open Source Software_, *6*(61), 3167. doi:10.21105/joss.03167 <https://doi.org/10.21105/joss.03167>, <https://doi.org/10.21105/joss.03167>. - Pattaro C, Fujii R (2022). _nephro: Utilities for Nephrology_. R package version 1.3, <https://CRAN.R-project.org/package=nephro>. - Pedersen T (2022). _ggforce: Accelerating 'ggplot2'_. R package version 0.4.1, <https://CRAN.R-project.org/package=ggforce>. - Pedersen T (2022). _patchwork: The Composer of Plots_. R package version 1.1.2, <https://CRAN.R-project.org/package=patchwork>. - Putter H (2015). _dynpred: Companion Package to "Dynamic Prediction in Clinical Survival Analysis"_. R package version 0.1.2, <https://CRAN.R-project.org/package=dynpred>. - R Core Team (2023). _R: A Language and Environment for Statistical Computing_. R Foundation for Statistical Computing, Vienna, Austria. <https://www.R-project.org/>. - Revell L (2012). “phytools: An R package for phylogenetic comparative biology (and other things).” _Methods in Ecology and Evolution_, *3*, 217-223. doi:10.1111/j.2041-210X.2011.00169.x <https://doi.org/10.1111/j.2041-210X.2011.00169.x>. - Robinson D, Hayes A, Couch S (2023). _broom: Convert Statistical Objects into Tidy Tibbles_. R package version 1.0.5, <https://CRAN.R-project.org/package=broom>. - Sarkar D (2008). _Lattice: Multivariate Data Visualization with R_. Springer, New York. ISBN 978-0-387-75968-5, <http://lmdvr.r-forge.r-project.org>. - Sarkar D, Andrews F (2022). _latticeExtra: Extra Graphical Utilities Based on Lattice_. R package version 0.6-30, <https://CRAN.R-project.org/package=latticeExtra>. - Schauberger P, Walker A (2023). _openxlsx: Read, Write and Edit xlsx Files_. R package version 4.2.5.2, <https://CRAN.R-project.org/package=openxlsx>. - Sievert C (2020). _Interactive Web-Based Data Visualization with R, plotly, and shiny_. Chapman and Hall/CRC. ISBN 9781138331457, <https://plotly-r.com>. - Sjoberg D, Whiting K, Curry M, Lavery J, Larmarange J (2021). “Reproducible Summary Tables with the gtsummary Package.” _The R Journal_, *13*, 570-580. doi:10.32614/RJ-2021-053 <https://doi.org/10.32614/RJ-2021-053>, <https://doi.org/10.32614/RJ-2021-053>. - Therneau T (2023). _A Package for Survival Analysis in R_. R package version 3.5-5, <https://CRAN.R-project.org/package=survival>. Terry M. Therneau, Patricia M. Grambsch (2000). _Modeling Survival Data: Extending the Cox Model_. Springer, New York. ISBN 0-387-98784-3. - Tierney N (2017). “visdat: Visualising Whole Data Frames.” _JOSS_, *2*(16), 355. doi:10.21105/joss.00355 <https://doi.org/10.21105/joss.00355>, <http://dx.doi.org/10.21105/joss.00355>. - Tierney N, Cook D (2023). “Expanding Tidy Data Principles to Facilitate Missing Data Exploration, Visualization and Assessment of Imputations.” _Journal of Statistical Software_, *105*(7), 1-31. doi:10.18637/jss.v105.i07 <https://doi.org/10.18637/jss.v105.i07>. - Urbanek S (2023). _rJava: Low-Level R to Java Interface_. R package version 1.0-10, <https://CRAN.R-project.org/package=rJava>. - Wickham H (2016). _ggplot2: Elegant Graphics for Data Analysis_. Springer-Verlag New York. ISBN 978-3-319-24277-4, <https://ggplot2.tidyverse.org>. - Wickham H (2022). _stringr: Simple, Consistent Wrappers for Common String Operations_. R package version 1.5.0, <https://CRAN.R-project.org/package=stringr>. - Wickham H (2023). _forcats: Tools for Working with Categorical Variables (Factors)_. R package version 1.0.0, <https://CRAN.R-project.org/package=forcats>. - Wickham H, Averick M, Bryan J, Chang W, McGowan LD, François R, Grolemund G, Hayes A, Henry L, Hester J, Kuhn M, Pedersen TL, Miller E, Bache SM, Müller K, Ooms J, Robinson D, Seidel DP, Spinu V, Takahashi K, Vaughan D, Wilke C, Woo K, Yutani H (2019). “Welcome to the tidyverse.” _Journal of Open Source Software_, *4*(43), 1686. doi:10.21105/joss.01686 <https://doi.org/10.21105/joss.01686>. - Wickham H, Bryan J (2023). _readxl: Read Excel Files_. R package version 1.4.3, <https://CRAN.R-project.org/package=readxl>. - Wickham H, François R, Henry L, Müller K, Vaughan D (2023). _dplyr: A Grammar of Data Manipulation_. R package version 1.1.2, <https://CRAN.R-project.org/package=dplyr>. - Wickham H, Henry L (2023). _purrr: Functional Programming Tools_. R package version 1.0.1, <https://CRAN.R-project.org/package=purrr>. - Wickham H, Hester J, Bryan J (2023). _readr: Read Rectangular Text Data_. R package version 2.1.4, <https://CRAN.R-project.org/package=readr>. - Wickham H, Miller E, Smith D (2023). _haven: Import and Export 'SPSS', 'Stata' and 'SAS' Files_. R package version 2.5.3, <https://CRAN.R-project.org/package=haven>. - Wickham H, Vaughan D, Girlich M (2023). _tidyr: Tidy Messy Data_. R package version 1.3.0, <https://CRAN.R-project.org/package=tidyr>. - Wilke C, Wiernik B (2022). _ggtext: Improved Text Rendering Support for 'ggplot2'_. R package version 0.1.2, <https://CRAN.R-project.org/package=ggtext>. - Wilke C, Wiernik B (2022). _gridtext: Improved Text Rendering Support for 'Grid' Graphics_. R package version 0.1.5, <https://CRAN.R-project.org/package=gridtext>. - Xie Y (2023). _knitr: A General-Purpose Package for Dynamic Report Generation in R_. R package version 1.43, <https://yihui.org/knitr/>. Xie Y (2015). _Dynamic Documents with R and knitr_, 2nd edition. Chapman and Hall/CRC, Boca Raton, Florida. ISBN 978-1498716963, <https://yihui.org/knitr/>. Xie Y (2014). “knitr: A Comprehensive Tool for Reproducible Research in R.” In Stodden V, Leisch F, Peng RD (eds.), _Implementing Reproducible Computational Research_. Chapman and Hall/CRC. ISBN 978-1466561595
